# RNA extraction from self-assembling peptide hydrogels to allow qPCR analysis of encapsulated cells

**DOI:** 10.1371/journal.pone.0197517

**Published:** 2018-06-04

**Authors:** Kyle A. Burgess, Victoria L. Workman, Mohamed A. Elsawy, Aline F. Miller, Delvac Oceandy, Alberto Saiani

**Affiliations:** 1 School of Materials, The University of Manchester, Manchester, United Kingdom; 2 Manchester Institute of Biotechnology, The University of Manchester, Manchester, United Kingdom; 3 School of Chemical Engineering and Analytical Sciences, The University of Manchester, Manchester, United Kingdom; 4 Division of Cardiovascular Sciences, The University of Manchester, Manchester, United Kingdom; Kyoto Daigaku, JAPAN

## Abstract

Self-assembling peptide hydrogels offer a novel 3-dimensional platform for many applications in cell culture and tissue engineering but are not compatible with current methods of RNA isolation; owing to interactions between RNA and the biomaterial. This study investigates the use of two techniques based on two different basic extraction principles: solution-based extraction and direct solid-state binding of RNA respectively, to extract RNA from cells encapsulated in four β-sheet forming self-assembling peptide hydrogels with varying net positive charge. RNA-peptide fibril interactions, rather than RNA-peptide molecular complexing, were found to interfere with the extraction process resulting in low yields. A column-based approach relying on RNA-specific binding was shown to be more suited to extracting RNA with higher purity from these peptide hydrogels owing to its reliance on strong specific RNA binding interactions which compete directly with RNA-peptide fibril interactions. In order to reduce the amount of fibrils present and improve RNA yields a broad spectrum enzyme solution—pronase—was used to partially digest the hydrogels before RNA extraction. This pre-treatment was shown to significantly increase the yield of RNA extracted, allowing downstream RT-qPCR to be performed.

## Introduction

**Fig 1:**
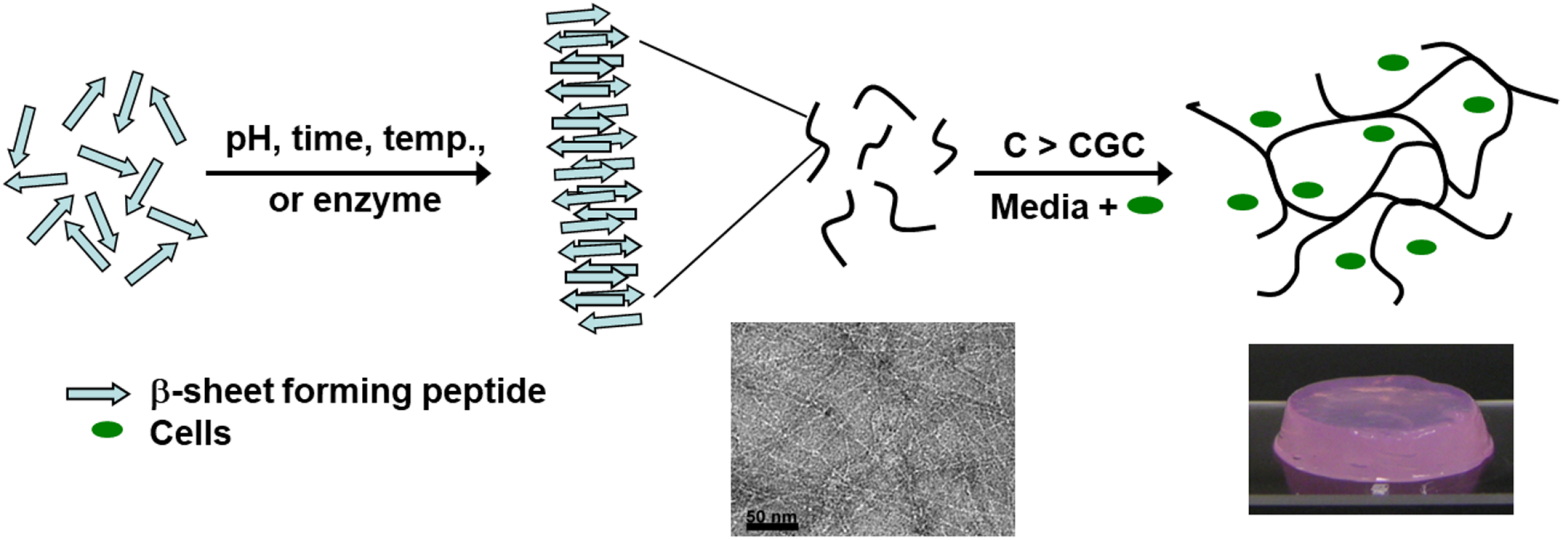
Schematic representation of the self-assembly and gelation pathway of β-sheet forming peptides. Short peptides are designed that self-assemble into β-sheet rich fibres that above a CGC entangle and/or associate to form 3D percolated networks swollen by water, i.e: hydrogels. Inserts: Typical TEM micrograph of nano-fibrillar network and photograph of hydrogel formed by these peptides.

In the past two decades, significant efforts have been made to design 3-dimensional scaffolds that mimic the extracellular matrix (ECM) for a variety of biomedical applications, such as tissue engineering and regenerative medicine. One such class of material are hydrogels, as these highly hydrated materials can be engineered to mimic the cellular niche.[1, 2] A variety of natural and synthetic building-blocks can be used to design hydrogels, one such block is the *de novo* designed self-assembling peptide. In the past decade a variety of designs have emerged that allow the synthesis of short peptides that self-assemble into fibrillar structures that, above a critical gelation concentration (CGC), associate and/or entangle to form 3D, percolated networks swollen by water; hence hydrogel.[3] Of particular interest is a family of amphipathic self-assembling peptides originally devised by Zhang’s group, the design of which is based on the alternation of hydrophilic and hydrophobic residues.[4, 5] These peptides have the ability to self-assemble into β-sheet nanofibrils which, above a CGC, entangle and/or associate to form hydrogels (Fig 1).

Zhang’s design has been the basis of many self-assembling peptide variants, some of which include: EAK16,[4, 6] KLD12,[7] RADA16 [8] and KFE8.[9]. The propensity for these peptides to self-assemble into β-sheet nanofibrils and the overall hydrogels’ properties are dependent on several factors, including: sequence, concentration, pH and ionic strength of the medium.[10–12] These β-sheet forming self-assembling peptide-based hydrogels have already proven a viable platform for cell culture [13–15], tissue engineering [16] and drug delivery [17]. Some variants are now readily available following the commercialisation of several systems, these include: Puramatrix (Corning), HydroMatrix (Sigma), and more recently PeptiGelDesign Technologies.

To determine the suitability of 3D matrices / scaffolds for cell culture, it is important to understand how the biomaterial influences cell behaviour. The biological response of cells to their surroundings is mirrored by changes in gene expression which can be observed using various techniques, including: northern blotting, quantitative polymerase chain reaction (qPCR) and microarray analysis. Regardless of the technique, it is crucial to first isolate good quality RNA. Most commercially available kits for RNA extraction rely on one of two basic principles, either: 1) solution-based, phase-separation using guanidinium thiocyanate, phenol and chloroform; or 2) direct, solid-state binding of RNA to a silica membrane within spin columns under conditions of high ionic strength.[18] The first method involves using a mixture of guanidine thiocyanate and phenol to dissolve RNA, DNA and protein on homogenization of the sample.[19] Chloroform is then added to form a biphasic emulsion comprising a hydrophobic, organic phase and a hydrophilic, aqueous phase that separates proteins from nucleic acids, respectively. When extraction is carried out under acidic conditions, RNA remains soluble and stays in the aqueous phase of the emulsion; whereas DNA and protein separate into the interphase and organic phase, respectively. RNA is then collected through precipitation of the aqueous phase with isopropanol. Alternatively, the second method involves solid-state binding of nucleic acids to a hydrated silica matrix using a specialized guanidine, thiocyanate-containing lysis buffer with high salt content.[18] Sodium ions break the hydrogen bonds between water and silica, and act as a cationic bridge to bind the negatively charged oxygen ions in silica with the negatively charged oxygen ions on nucleic acids. Other contaminants might adsorb to the surface, but do so with weaker affinity and are thus removed through subsequent wash steps using a competitive agent. RNA is then eluted by changing to a solution of low ionic strength.

Isolating sufficient amounts of good quality RNA from cells encapsulated within hydrogels has proven difficult with gel contaminants hindering downstream applications, such as reverse-transcription-qPCR (RT-qPCR).[20, 21] Optimisation of current RNA extraction processes has already shown to be necessary for several different types of hydrogels, both of a natural composition: agarose,[20–22] alginate,[21] chitosan,[22, 23] gelatin [21] and gellan;[20] as well as of a synthetic nature: polyethylene glycol.[24] The low yield of RNA isolated from encapsulated cells is thought to be a result of ionic complexing between negatively charged RNA and positively charged regions of the matrix.[22] No one technique has yet shown to be most effective for isolating a good yield of pure RNA from all types of hydrogel. Instead, differential optimisation of current RNA extraction methods is required for each type of hydrogel depending on the matrix composition.

Typically β-sheet forming self-assembling peptides tend to contain multiple protonated residues. The overall charge carried by the peptide/peptide fibre is related to the excess cationic residues present in the sequence. If the peptide contains the same number of cationic (e.g: lysine and arginine) and anionic (e.g.: glutamic acid and aspartic acid) residues then the peptide/peptide fibre will be neutral at pH 7. Alternatively if there are a higher number of cationic residues then the peptide/peptide fibre will carry a net positive charge. It is therefore of little surprise that these hydrogels also interfere with the RNA extraction process. In the present study we compare and optimise the extraction of RNA from human endothelial kidney cells (HEK293) encapsulated in four different β-sheet forming self-assembling peptide-based hydrogels with varying net positive charges: PGD-Alpha1 (neutral), PGD-Alpha2 (medium net positive charge), PGD-AlphaProB (high net positive charge) and PGD-AlphaProC (high net positive charge) at pH 7. These peptide hydrogels were selected because they offer a range of mechanical properties and relative net positive charges at neutral pH which we have shown recently can affect cell behaviour.[25] This set of hydrogels allowed us to study the effect of electrostatic interactions between RNA and self-assembling peptides and/or peptide fibres on the RNA extraction process. Two extraction kits were used: one solution-based, TRI Reagent^®^ (Sigma-Aldrich); and one column-based, RNeasy Mini Kit^®^ (Qiagen). The effect of first enzymatically degrading the hydrogels prior to RNA extraction was also investigated. The extracted RNA was evaluated in terms of: yield and purity, using UV spectroscopy; integrity, using electrophoresis; and quality, using RT-qPCR analysis of common housekeeping genes to assess its suitability in downstream applications.

## Materials and methods

### Materials

All materials and reagents were purchased from Thermo Fisher Scientific (Loughborough, UK), unless stated otherwise. The following four peptide hydrogels were purchased from PeptiGel*Design* Technologies (Alderley Edge, UK): PGD-Alpha1 (neutral at pH 7), PGD-Alpha2 (medium net positive charge at pH 7), PGD-AlphaProB (high net positive charge at pH 7) and PGD-AlphaProC (high net positive charge at pH 7). Peptide solutions corresponding to each hydrogel formulation for electrophoresis were kindly supplied by PeptiGel*Design* Technologies. Additional information on the hydrogels is available on request to PeptiGel*Design* Technologies (www.peptigeldesign.com).

### Cell culture

Human endothelial kidney cells (HEK293) (HEK293A, R70507; Thermo Fisher Scientific) were maintained under standard cell culture conditions in Dulbecco’s modified Eagle’s medium (DMEM) supplemented with 10% fetal bovine serum (FBS), 1% penicillin/streptomycin solution and MEM non-essential amino acids solution (1 ×). At 80%–90% of confluence, cells were sub-cultured using TrypLE cell dissociation reagent, transferred to a Falcon tube and centrifuged to remove the TrypLE solution. The cell pellet was then re-suspended in fresh medium and adjusted to the required cell concentration before being re-seeded onto tissue culture plastic. For 3D cell encapsulation, 90 μL of each hydrogel was first pipetted using a precision positive displacement microliter pipette (Microman^®^; Gilson, Bedfordshire, UK) into 24-well cell culture inserts (ThinCert^™^; Greiner, Stonehouse, UK), to obtain a final cylindrical hydrogel with a height of ~1.5 mm. Cells were cultured to 80% confluence, detached using TrypLE cell dissociation reagent and re-suspended to achieve a stock solution of 400 × 10^6^ cells mL^−1^. A 10 μl aliquot of the cell stock solution was physically mixed into the hydrogel using the tip of a 20 μL pipette, thereby producing a hydrogel of 100 μL containing ~ 4 × 10^6^ cells. Media was then placed in and around the insert and the hydrogels were placed in an incubator at 37 °C in a 95% humidified atmosphere (20% O_2_) with 5% CO_2_. By incubating the hydrogels with media all hydrogels were subsequently buffered to pH 7. For cell-only controls, cells were treated as above but re-suspended in 90 μL of Dulbecco’s phosphate-buffered saline (dPBS) (without Ca^2+^ and Mg^2+^; Sigma-Aldrich, Dorset, UK) with no media incubation.

#### RNA extraction

RNA was extracted using the following commercial kits: RNeasy Mini Kit^®^ (Qiagen, Manchester, UK) or TRI Reagent^®^ (Sigma-Aldrich, Dorset, UK). Briefly, the hydrogels were seeded with ~ 4 × 10^6^ cells and incubated for 30 minutes. The cell culture medium was then removed before each hydrogel was washed three times in dPBS for 5 minutes. When using the kits alone (TRI Reagent method and RNeasy Mini Kit (MK) method), the hydrogels were then suspended directly in either 1 mL of TRI Reagent^®^ or 350 μL of RNeasy Lysis Buffer^®^ and homogenised at speed 6 (out of 10) for 15 seconds using an automated homogeniser (IKA RW16 basic; Sigma-Aldrich, Dorset, UK). RNA was then isolated according to manufacturer’s instructions. For the combined TRI reagent^®^ and RNeasy Mini Kit^®^ method (TRI Reagent + RNeasy MK method), following the washing step the hydrogels were homogenised, as described, in 1 mL of TRI Reagent^®^. Then 0.2 mL of chloroform was added and the solution was incubated at room temperature for 15 minutes. The solution was then centrifuged at 12,000 × g for 15 minutes at 4 °C. The aqueous phase was then removed and combined with an equal volume of 70% ethanol and then applied to the RNeasy MK spin columns, as per manufacturer’s recommendations. For all methods, the RNA was eluted in 30 μL of RNase-free water. For the enzymatic degradation, each hydrogel was washed as previously mentioned, removed from the insert and placed in a microcentrifuge tube. Then, 4 volumes of pronase solution (10 mg mL^−1^ stock solution prepared in DNase and RNase-free water) were added to the hydrogel, triturated and placed in a 37 °C water bath for 5 minutes with gentle agitation every minute. The hydrogel solution was then added directly to the RNeasy^®^ lysis buffer, homogenised and extracted as described above.

### Determination of RNA concentration, purity and integrity

For all samples and controls, RNA was extracted from 4 × 10^6^ cells and re-suspended in 30 μL of water. The concentrations of RNA extracted from all samples were calculated using UV spectroscopy by measuring the absorbance at 260 nm (A_260_). The value was calculated using the Lambert-Beer law based on absorbance at 260 nm (1 A_260_ unit = 40 mg mL^−1^ RNA). For each sample, a 1 μL aliquot was taken and measured using a spectrophotometer (Nanodrop^™^ 1000; NanoDrop Technologies). From the absorbance spectra (220–350 nm), the A_260_/A_280_ and A_260_/A_230_ ratios were used to assess the purity of RNA. Ratio values of ≥ 1.8 for A_260_/A_280_ and ≥ 2 for A_260_/A_230_ are an acceptable indicator of good quality RNA.[26–28] The A_260_/A_220_ ratio was used to estimate the level of peptide contamination. Three independent samples were measured with the data shown as mean ± standard error of mean (SEM). To determine RNA integrity, each RNA sample (100 μg) was run on a TapeStation automated electrophoresis analysis system (2200 TapeStation; Agilent Technologies, Stockport, UK). Separation profiles composed of intact 28S (4.5 kb), 18S (1.9 kb) and small RNA species were compared. The RNA integrity number (RIN) values were determined using the TapeStation analysis software. Three independent samples were measured for each sample.

#### RNA gel electrophoresis

RNA was isolated from HEK293 cells using the TRI Reagent^®^ method, described previously. Then 1 μg of total RNA was incubated with solutions of each peptide at different molar concentrations, before then being run on a non-denaturing agarose (0.8%) gel. Briefly, 5 mM of lyophilized peptide, corresponding to each hydrogel formulation, was dissolved in RNase-free water, sonicated for 1 hour and left overnight at 4 °C. A serial dilution was created for each peptide sequence by diluting the samples 1:5 with RNase-free water. Then, equal volumes of RNA and peptide solution were combined to create a 30 μL solution at the following peptide concentrations: 2.5 mM, 0.5 mM, 0.1 mM, 0.02 mM and 0.004 mM; each containing 1 μg of RNA. The RNA-peptide solutions were incubated on ice for 30 minutes, mixed with DNA loading buffer (6 ×) and loaded into the well. Gel electrophoresis was performed at 75 V for 40 minutes in 0.5 × TBE running buffer. For reference, an RNA-only sample was run to show the expected bands and relative intensities (positive control). A peptide-only control, at the highest concentration (2.5 mM), was also run (negative control). The agarose gel was prepared in 0.5 × TBE running buffer using molecular grade agarose (Bioline, London, UK). The agarose gel and running buffer were diluted using diethyl pyrocarbonate (DEPC)-treated water. RNA was stained with SYBER^®^ Safe (10,000 ×) which was incorporated into the gel before casting. The gel was imaged using a Chemidoc imaging system (Bio-Rad, Hertfordshire, UK). All equipment was washed according to the following protocol: wash with 1% sodium dodecyl sulphate (SDS) in distilled water, then with 100% ethanol, then incubate with 3% hydrogen peroxide (H_2_O_2_) in distilled water for 10 minutes and finally rinse with DEPC-treated water.

### Reverse transcription quantitative polymerase chain reaction (RT-qPCR)

For RT-qPCR, total RNA was isolated, as previously described, and either 0.5 μg (RNeasy Mini Kit^®^/ TRI Reagent^®^) or 1 μg (post-enzymatic degradation) of RNA from each sample was converted into cDNA. Briefly, total RNA was treated with amplification grade DNase I (Sigma Aldrich, Dorset, UK) and transcribed into cDNA using a high capacity RNA-to-cDNA kit, supplemented with RNase inhibitor, as per manufacturer’s instructions. For qPCR, cDNA was diluted 1: 1 (0.5 μg) or 1:3 (1 μg) with RNase-free water and the Brilliant III Ultra-Fast SYBR^®^ QPCR kit (Agilent Technologies, Stockport, UK) was used following a two-step RT-qPCR protocol: enzyme activation for 3 minutes at 95 °C, followed by 40 cycles of denaturation for 5 seconds and annealing/elongation for 25 seconds at 60 °C. All reactions were performed in triplicate using pre-designed primers (QuantiTect; Qiagen, Manchester, UK) for 5 common housekeeping genes: GAPDH, encoding glyceraldehyde 3-phosphate dehydrogenase; RPL13A, encoding ribosomal protein L13A; ACTB, encoding β-actin; B2M, encoding β2-microglobulin; and RRN18s, encoding 18S ribosomal RNA. All qPCR reactions were performed on an ABI 7500 Fast System (Applied Biosystems). Threshold cycle (Ct) values were determined by using the Sequence Detection System software. The expected product sizes were confirmed by melting temperature (T_m_) analysis in the range 60–95 °C; all samples produced identical, overlapping melting curves with a single peak at the expected T_m_ value.

### Enzymatic digestion of hydrogels

Enzymes were prepared as per manufacturer recommendations at the following concentrations: Papain: 6.25 mg mL^−1^ in buffer composed of 0.2 M sodium phosphate buffer, 100 mM sodium acetate, 10 mM EDTA (di-sodium salt), 5 mM cysteine HCl; Pronase: 10 mg mL^−1^ in HPLC grade H_2_O; Proteinase K: 400 μg mL^−1^ in 50 mM TrisHCl and 2.5 mM CaCl_2_ and Thermolysin: 1 mg mL^−1^ in 50 mM TrisHCl and 0.5 mM CaCl_2_. Trypsin was used as supplied by the manufacturers. Peptide hydrogels (250 μL) were pipetted into 12-well ThinCerts^™^, covered with medium (as used in cell culture experiments) and allowed to incubate for 24 hours in a 37 °C / 5% CO_2_ humidified incubator. Hydrogels were removed from the inserts and added to 1 mL of enzyme solution. Samples were incubated at 37 °C and triturated with a Pasteur pipette for 10 seconds every minute. Hydrogel samples were collected after 5 minutes of incubation with the enzyme and were diluted to 1mg/ml^−1^ using H_2_O:CH_3_CN (1:1 v/v) HPLC grade solvent mixture (80% H_2_O / 20% CH_3_CN with 1% TFA). HPLC was used to measure the amount of non-degraded peptide in each sample. An analytical scale Phenomenex Jupiter 4 μm Proteo column 90 A° (250 × 4.66 mm) was used with a flow rate of 1 mL min^−1^. The column was equilibrated in 90% H_2_O / 10% CH_3_CN with 0.05% TFA, followed by a 200 μL sample injection. The elution gradient used went from 90% H_2_O / 10% CH_3_CN to 30% H2O / 70% CH_3_CN (all solvents contained 0.05% of TFA) over 45 minutes. The percentage of non-degraded peptide was calculated using the following equation:

%non-degradedpeptide=(AUPt/AUPc)×100 (1)

where, *AUPt* and *AUPc* are the areas under the HPLC peaks, as calculated by Chromeleon software, for the sample treated 5 minutes with the enzyme and the untreated control sample, respectively.[29] At least three repeat experiments were undertaken. For the untreated control, the hydrogel was re-suspended in the same volume of water as enzyme solution.

### Statistical analysis

The RNA yield and purity were calculated from three independent samples, and the RT-qPCR experiments were also carried out from three independent samples and run in triplicate. The mean and SEM were calculated from the three independent repeats, where the averages of the RT-qPCR triplicates were used for each biological repeat. To compare the mean values, a two sample t-test was performed and statistical significance was determined when: *, *P* ≤ 0.05; **, *P ≤ 0*.01; ***, *P ≤ 0*.*001*, compared to the control. For hydrogel degradation, the mean and SEM were calculated from three independent repeats and statistical significance was determined using the Kruskal-Wallis H-test.

## Results and discussion

### Peptide / Peptide fibril—RNA interactions

**Fig 2:**
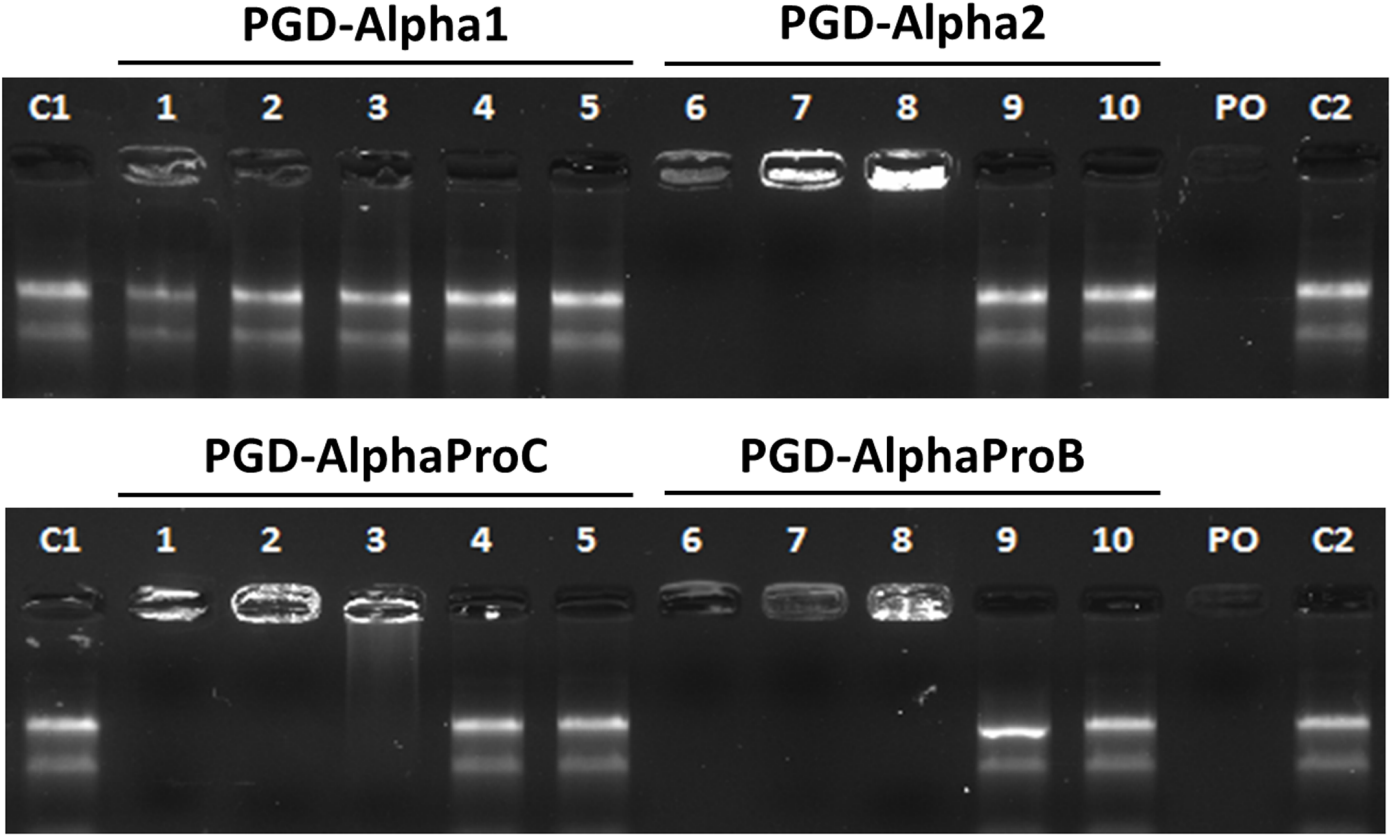
Native agarose gel electrophoresis traces of total RNA (1 μg) following incubation with peptide solutions of PGD-Alpha1, PGD-Alpha2, PGD-AlphaProB and PGD-AlphaProC at peptide concentrations of 2.5 mM (1+6), 0.5 mM (2+7), 0.1 mM (3+8), 0.02 mM (4+9) or 0.004 mM (5+10). Total RNA-only (C1+C2) and peptide-only (PO) samples were run as controls. The total RNA-only controls highlight the presence of two bands correlating to the 28S and 18S rRNA.

β-sheet forming self-assembling peptides typically contain polar residues that will carry positive (e.g: lysine & arginine) or negative (e.g.: aspartic acid & glutamic acid) charges around neutral pH. In addition, if unprotected and unmodified these peptides will also have a carboxyl and an amino end-group, which will also carry a negative and a positive charge at pH 7, respectively. As a result, if the number of positively charged groups is higher than the number of negatively charged groups on the peptide, then both the peptide monomer and its fibrillar aggregate (peptide fibril) will carry a net positive charge around neutral pH. Therefore, the peptide and/or peptide fibril can interact with the negatively charged RNA and interfere with its extraction process. To demonstrate that RNA interacts electrostatically with the peptide and/or peptide fibril of our hydrogels, gel electrophoresis was performed on RNA that had been pre-incubated with solutions of peptide from each hydrogel formulation at different molar concentrations (all below the CGC of the peptides). The hypothesis being; if the peptide and/or peptide fibrils interact with RNA then it will affect the RNA mobility and cause a shift in the expected band sizes. As such, RNA-peptide samples were run on a non-denaturing agarose gel and the separation profiles were compared to an RNA-only control. As can be seen from Fig 2, the RNA-only control separated out into two distinct bands which represent the 28S and 18S ribosomal RNA (rRNA), as expected. No smaller RNA species were visible and the separation profile was not smeared; indicating the RNA was intact. When the RNA was pre-incubated with solutions of peptide, only RNA incubated with PGD-Alpha1, the neutral peptide, was able to separate out into two distinctive bands at all concentrations investigated. For the highest concentration used, 2.5 mM, some RNA appeared to be present in the well but still a separation profile with distinct bands for the 28S and 18S rRNA was obtained. However, for PGD-Alpha2, PGD-AlphaProB and PGD-AlphaProC, the three peptides that carry a positive net charge, the RNA remained trapped within the well and failed to separate out along the gel when incubated with peptide solutions at concentrations ≥ 0.1 mM. For concentrations ≤ 0.02 mM, the RNA separated out into two clear bands comparable to the RNA-only control.

RNA-peptide molecular complexing through electrostatic interactions has been observed with signal peptides, which are characterised by having a positively charged region.[30] The fact that the presence of positively charged peptide traps the RNA within the well of the agarose gel, not just retard RNA migration, suggests that it is part of a large structure with a molecular size larger than that of the agarose gel pores. These results clearly show that there is a strong electrostatic interaction between the RNA and the peptides fibrils (assembled peptides), rather than the peptide molecules (non-assembled peptides). The fact that at very low concentrations, below the critical self-assembly concentration (CSAC) of this family of peptides [31–33], the RNA is able to run through the gel unhindered seems to support this conclusion, indeed, below the CSAC fibrils do not form. These results show that the presence of peptide molecules (non-assembled peptides) does not interfere with RNA mobility pointing towards the absence of molecular complexing between the RNA and these peptide molecules. As mentioned above, for PGD-Alpha1, the neutral peptide, some RNA retention in the well is observed at the highest peptide concentration used suggesting weak non-electrostatic interactions between peptide fibres and RNA in this case. It should be kept in mind that when using hydrogels the peptide concentrations will be significantly higher, and therefore, as shown below, weak interactions, in addition to electrostatic interactions, will also interfere with the RNA separation process.

### RNA extraction from peptide hydrogels

**Fig 3:**
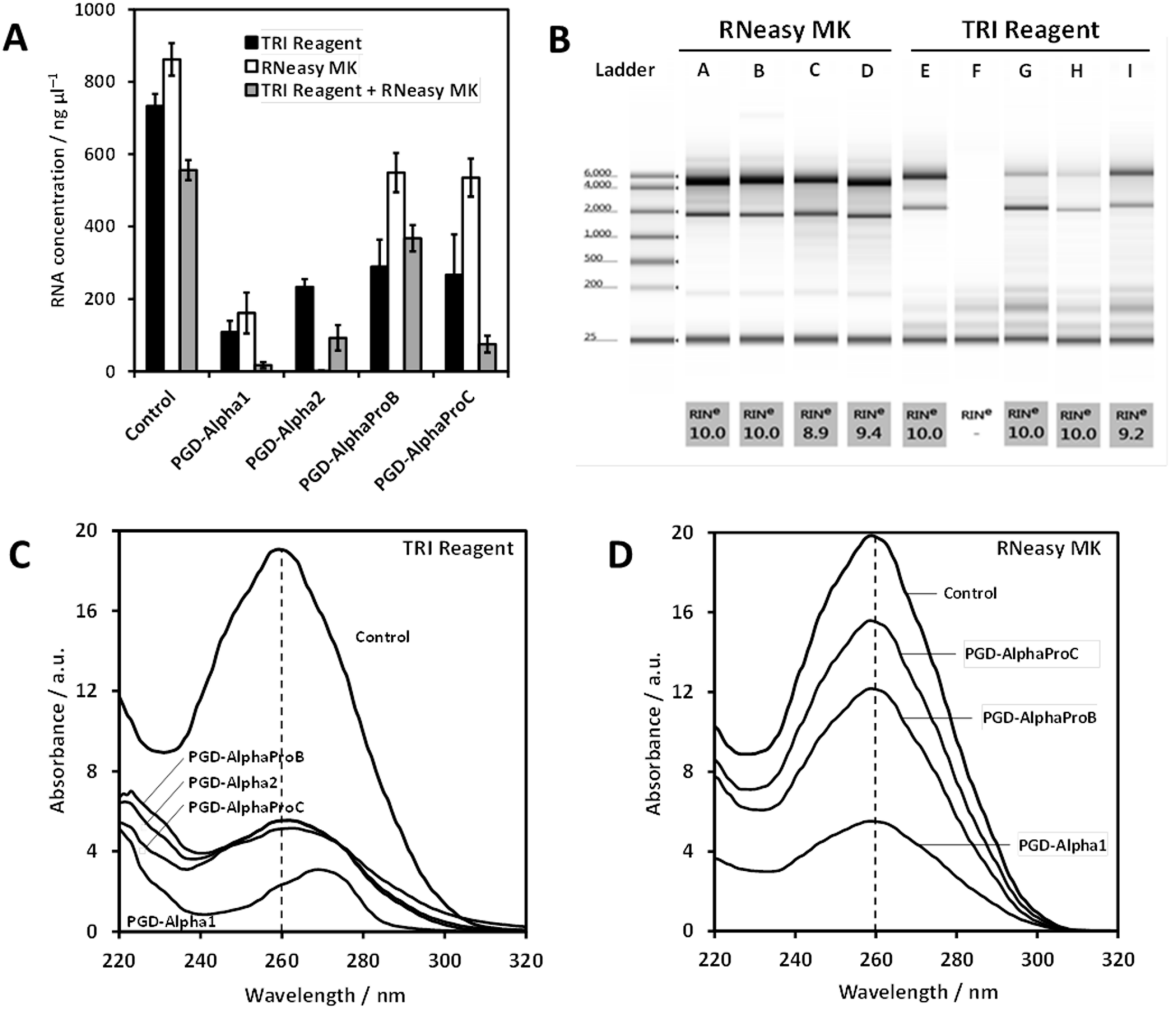
A) Concentration of RNA extracted from HEK293 cells encapsulated in the four peptide hydrogels and cell-only controls using the three methods (see text for details). **B** Representative electrophoresis traces of total RNA extracted from cells encapsulated in the four peptide hydrogels and cell-only controls using the Tri Reagent and RNeasy MK methods and corresponding RIN values: cell-only controls (A+E), PGD-Alpha1 (B+F), PGD-Alpha2 (G), PGD-AlphaProB (C+H) and PGD-AlphaProC (D+I). C & D) Representative UV spectra for RNA samples extracted using the TRI Reagent (C) and the RNeasy MK (D) methods from the four peptide hydrogels and cell-only control.

Attempts were made to extract RNA from cells using two commercial kits: one solution-based, TRI Reagent^®^ (Sigma-Aldrich), and one column-based, RNeasy Mini Kit^®^ (Qiagen). Three different protocols were used: 1) TRI Reagent method (TRI Reagent^®^ kit only), 2) RNeasy MK method (RNeasy Mini Kit^®^ only) and 3) TRI Reagent + RNeasy MK method (TRI Reagent^®^ kit followed by RNeasy Mini Kit^®^). This latter method was designed to test whether the RNeasy MK method could improve the purity of RNA extracted using the TRI Reagent method. Cells were encapsulated within the peptide hydrogels that were then allowed to set in media for 30 minutes. This time was selected in order to minimise changes in cell gene expression between the hydrogels. Cell-only 3D controls (cells suspended in PBS for 30 min.) were carried out for each extraction method. Both TRI Reagent and RNeasy MK methods were capable of isolating comparable concentrations of pure RNA from the controls. As expected for the TRI Reagent + RNeasy MK method a lower concentration of pure RNA was extracted compared to either method used separately (Fig 3A and Table 1).

**Table 1:** A_260_/A_220_, A_260_/A_230_ and A_260_/A_280_ ratios for all RNA samples isolated from the four hydrogels and cell-only control using the three different methods. Data is shown as mean ± SEM of three independent samples. Pure RNA has typically an A_260_/A_280_ ratio ≥ 1.8 and A_260_/A_230_ ratio ≥ 2. (‘-’, no measurable amount of RNA was extracted).

	TRI Reagent method			RNeasy MK method			TRI Reagent + RNeasy MK method		
	A_260_/A_280_	A_260_/A_230_	A_260_/A_220_	A_260_/A_280_	A_260_/A_230_	A_260_/A_220_	A_260_/A_280_	A_260_/A_230_	A_260_/A_220_
Control	1.94±0.01	1.96±0.14	1.47±0.12	2.07±0.01	2.21±0.01	1.91±0.02	2.08±0.01	2.07±0.15	1.68±0.17
PGD-Alpha1	1.76±0.04	0.79±0.15	0.48±0.08	2.05±0.02	0.94±0.45	0.95±0.31	1.64±0.13	0.35±0.10	0.22±0.09
PGD-Alpha2	1.67±0.08	0.74±0.21	1.03±0.14	-	-	-	2.02±0.05	1.25±0.09	0.88±0.12
PGD-AlphaProB	1.52±0.13	0.94±0.08	1.02±0.27	2.09±0.02	2.14±0.08	1.69±0.06	2.05±0.01	1.92±0.11	1.90±0.11
PGD-AlphaProC	1.90±0.02	1.27±0.12	1.12±0.30	2.06±0.02	2.14±0.05	1.83±0.06	1.95±0.07	1.12±0.31	0.83±0.18

UV absorbance at 260 nm (Fig 3C and 3D) was used to determine the concentration of RNA extracted (Fig 3A). For the TRI Reagent method, there was no significant difference between the concentrations of RNA isolated from PGD-Alpha2 (233 ± 22 ng μL^−1^), PGD-AlphaProC (266 ± 112 ng μL^−1^) and PGD-AlphaProB (290 ± 74 ng μL^−1^). The lowest yield of RNA was obtained for PGD-Alpha1 (108 ± 31 ng μL^−1^), approximately half the concentration obtained from the other three hydrogels. In all cases, the RNA concentrations were much lower than that of the cell-only control (733 ± 34 ng μL^−1^). For the RNeasy MK method, there was little difference between the amount of RNA extracted from PGD-AlphaProC (535 ± 53 ng μL^−1^) and PGD-AlphaProB (549 ± 54 ng μL^−1^), although the concentration of RNA obtained was almost double that of the TRI Reagent method. No RNA was extracted from PGD-Alpha2 when using RNeasy MK method. For PGD-Alpha1 the concentration of RNA obtained (161 ± 57 ng μL^−1^) was similar to the amount extracted using the TRI Reagent method but much lower than that extracted from PGD-AlphaProC and PGD-AlphaProB. For all four hydrogels the concentration of RNA extracted using the RNeasy MK method was lower than the cell-only control (862 ± 45 ng μL^−1^). For the TRI Reagent + RNeasy MK method, as expected the relative concentration of RNA obtained for each hydrogel was related to the amount of RNA that could be extracted when using the TRI Reagent method. Except for PGD-AlphaProB, for which no statistically significant difference could be observed, the concentration of RNA obtained was much lower using the TRI Reagent + RNeasy MK method than the TRI Reagent method only.

RNA purity was estimated by measuring the standard absorbance ratios A_260_/A_280_ and A_260_/A_230_ (Fig 3C and 3D). The peptide backbone absorbs at ≥ 220 nm [34], therefore the A_260_/A_220_ ratio was also used to estimate the level of peptide contamination. The results obtained have been compiled in Table 1. For pure RNA, typically values ≥ 1.8 for A_260_/A_280_, and ≥ 2 for A_260_/A_230_ are expected.[28]

When using the TRI Reagent method, the purity of the RNA extracted from the cells encapsulated in all four hydrogels was lower than the purity of the RNA extracted from the cell-only control. In particular the A_260_/A_220_ ratio was found to be low compared to the cell-only control, which suggests that significant amounts of peptide are carried through using this method. As mentioned in the introduction, the TRI Reagent method is based on the production of a biphasic emulsion comprising a hydrophobic, organic phase and a hydrophilic, aqueous phase that separate proteins from nucleic acids, respectively. When the extraction is carried out under acidic conditions, RNA remains soluble and stays in the aqueous phase. However, the problem when dealing with small monomeric peptides is that their water solubility is higher than that of proteins and, in our case, the solubility of the peptides used are also higher at acidic pH. Moreover, the complexation of the RNA with self-assembled peptide fibrils, which are expected—like proteins—to separate into the organic phase, will result in the sequestration of the RNA in the organic phase. We speculate that these two effects explain the low yield and low purity of the RNA obtained using the TRI Reagent method. In addition, close inspection of the UV spectra (Fig 3C) clearly shows that, for all hydrogel samples, there is the presence of a strong absorption band centred around 270 nm which, most likely indicates that phenol has been carried though the extraction process.[19] Since this absorption is not observed with the cell-only control, it suggests that these peptides may also interact with this contaminant and promote its solubilisation within the aqueous phase. From the spectra, it can clearly be seen that this band at 270 nm overlaps with the RNA band (nucleic acids) at 260 nm, probably resulting in the above concentrations of extracted RNA (calculated based on the intensity of the 260 nm band) being an overestimate of the true RNA concentration for the TRI Reagent method.

The RNeasy MK extraction method is based on the strong binding of the RNA to a silica matrix mediated by sodium ions (bridging effect). In this case electrostatic interactions play a key role in the separation process. When using this method, the purity of RNA extracted from cells encapsulated in the two systems carrying the highest positive net charge—PGD-AlphaProB and PGD-AlphaProC—was comparable to the cell-only control and suggests a stronger binding affinity of RNA to the matrix rather than to the peptide fibrils. However, the lower yields compared to the cell-only control still suggests that some RNA is lost and washed away; most likely a result of peptide fibrils interfering with the binding of RNA to the silica matrix. For PGD-Alpha1, the neutral peptide, contaminated RNA was extracted. The A_260_/A_220_ ratio was found to be significantly lower than the cell-only control suggesting that peptide was the main contaminant and that it binds strongly to the matrix. We hypothesise that this strong interaction between the peptide / peptide fibrils and the matrix interferes with and prevents RNA from binding to the matrix. As a result, a very low RNA yield was obtained for this hydrogel. For PGD-Alpha2, no RNA could be extracted using this method suggesting that this peptide also prevents RNA from binding to the matrix.

For PGD-AlphaProB, comparably pure RNA was also extracted using the TRI Reagent + RNeasy MK method. For PGD-AlphaProC, the prior use of the TRI Reagent method resulted in contamination that was not observed when using the RNeasy MK method alone. Since RNA extracted from PGD-Alpha1, PGD-Alpha2 and PGD-AlphaProC using the TRI Reagent + RNeasy MK method still contained contaminants, and also resulted in lower RNA yields, it was not investigated further.

To examine the integrity of the RNA extracted using the TRI Reagent and RNeasy MK methods, samples were analysed using electrophoresis (Fig 3B). All control samples were observed to produce separation profiles composed of intact 28S (5 kb) and 18S (2 kb) rRNA. To quantifiably compare the integrity of the RNA samples, the RNA integrity number (RIN) was assigned (automatically, by the TapeStation software) based on the entire electrophoretic trace. RIN values range from 1 to 10, the latter value depicting intact RNA. A RIN value of 10 was assigned to the RNA extracted from the controls using both methods. Except for PGD-Alpha2 RNeasy MK method, for which no RNA was obtained, and for PGD-Alpha1 TRI Reagent method, all other RNA samples extracted from encapsulated cells were observed to produce separation profiles composed of intact RNA species with RIN values between 9 and 10 (Fig 3B) indicating that the RNA extracted was intact. For PGD-Alpha1 TRI Reagent method, no RNA was detected probably due to the low amount and low purity of the RNA extracted. Indeed, as can be seen from the UV spectrum of this sample (Fig 3C), the 260 nm band is visible as a small shoulder on top of the 270 nm absorption band; clearly showing that the RNA concentration obtained from the 260 nm band intensity significantly overestimates the actual amount of RNA extracted.

**Fig 4:**
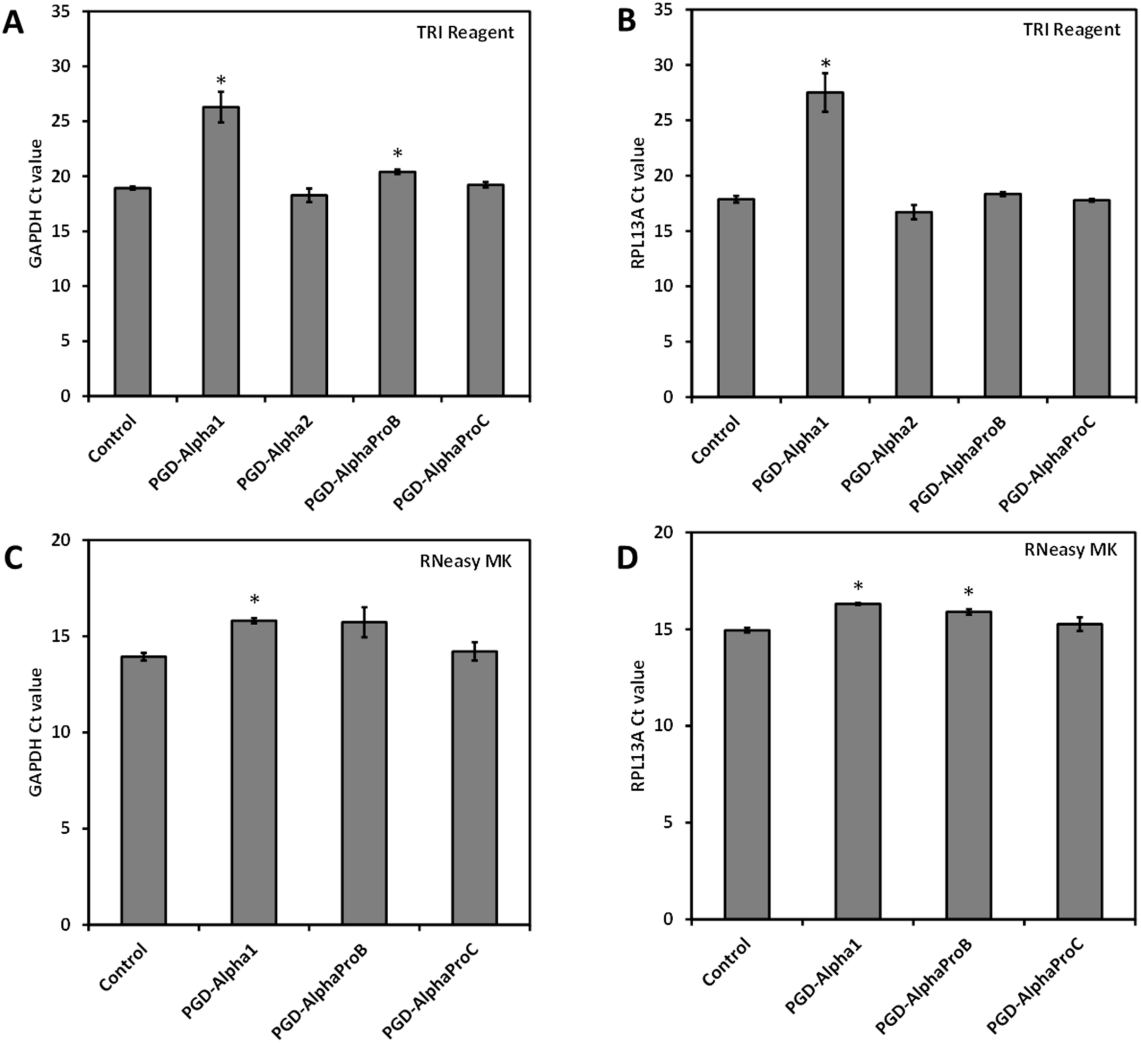
Ct values obtained for RT-qPCR performed using RNA extracted from the four peptide hydrogels as a template. RNA isolated from cells in suspension was used as a control. The RNA extracted using either the TRI Reagent method (A-B) or RNeasy MK method (C+D) was used as templates for the amplification of two housekeeping genes: GAPDH (A+C) and RPL13A (B+D). The cycle threshold (Ct) value was determined for three independent samples measured in triplicate (or two independent samples for PGD-Alpha1 using the RNeasy method). Data is shown as mean ± SEM. The mean values were compared to the non-encapsulated cell controls using a t-test; *, P ≤ 0.05.

To assess the quality of RNA, cDNA was prepared from all samples of RNA extracted using both methods (TRI Reagent and RNeasy MK). The expression levels (Ct values) of two commonly used endogenous control genes; glyceraldehyde 3-phosphate dehydrogenase (GAPDH) and ribosomal protein L13A (RPL13A), were analysed using RT-qPCR and compared to values obtained from the cell-only controls (Fig 4). A low Ct value (typically < 20) correlates with higher gene expression. For RNA extracted from cell-only controls using both methods Ct values < 20 were indeed obtained showing high RNA quality and high amplification efficiency.

RNA extracted from cells encapsulated in PGD-AlphaProB and PGD-AlphaProC showed no difference in amplification efficiency compared to the controls for either extraction method. This data indicates that, in both cases, the amount of RNA extracted and its purity was high enough to perform RT-qPCR. For PGD-Alpha1, Ct values similar to the control and < 20 were obtained only when RNA was extracted using the RNeasy MK method. For RNA extracted from PGD-Alpha1 using the TRI Reagent method, a Ct value > 20 was obtained; confirming that the concentration of RNA for this sample was an overestimate and so less RNA was converted into cDNA for use in the RT-qPCR reaction. Finally for PGD-Alpha2, the Ct values obtained for RNA extracted using the TRI Reagent method were similar to the control sample. No results are shown for PGD-Alpha2 using the RNeasy MK method as no RNA could be extracted. These results suggest, in particular for the TRI Reagent method, that the peptide carried through the extraction process does not interfere with RT-qPCR reaction suggesting, as above, that there is no or weak complexation at the molecular level between the RNA and these peptides. These findings also clearly indicate that the amount of RNA extracted, but more importantly the purity of RNA is critical in generating reliable and reproducible RT-qPCR data.

### Enzymatic proteolysis of hydrogels

As discussed above, we hypothesised that the interaction between RNA and the peptide fibrils was the main reason for the low RNA yields obtained. In an effort to reduce the amount of fibrils present in the samples, enzymes were used to attempt to proteolytically degrade the peptide monomers corresponding to each of the four hydrogels investigated.

**Fig 5:**
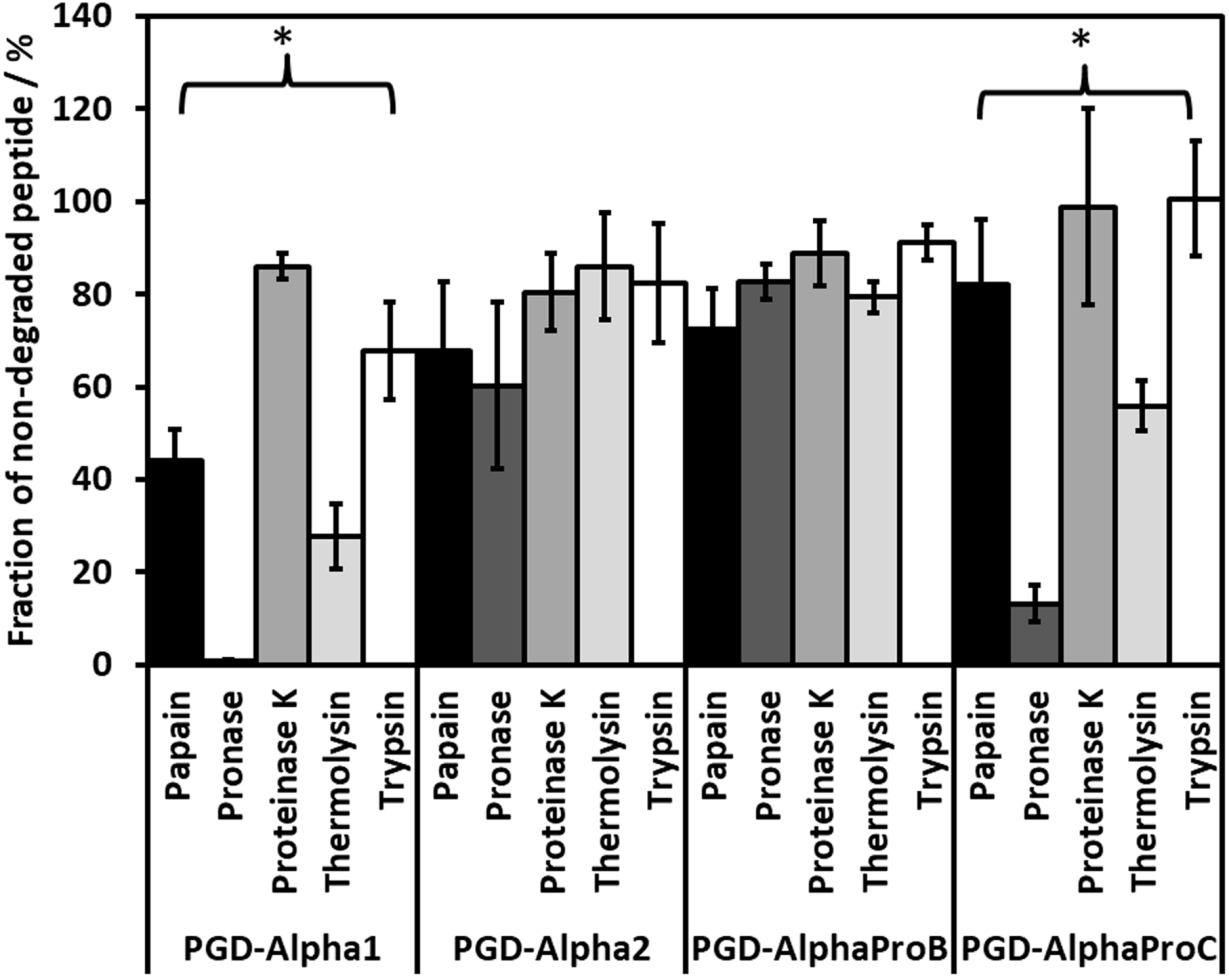
Percentage of non-degraded peptide monomer present after 5 minutes of enzyme degradation using five commercially available enzyme mixtures. The percentage of non-degraded peptide was determined by HPLC using non-treated hydrogel samples as controls. The results show the mean ± SEM of three independent samples. *, P ≤ 0.05.

**Fig 6:**
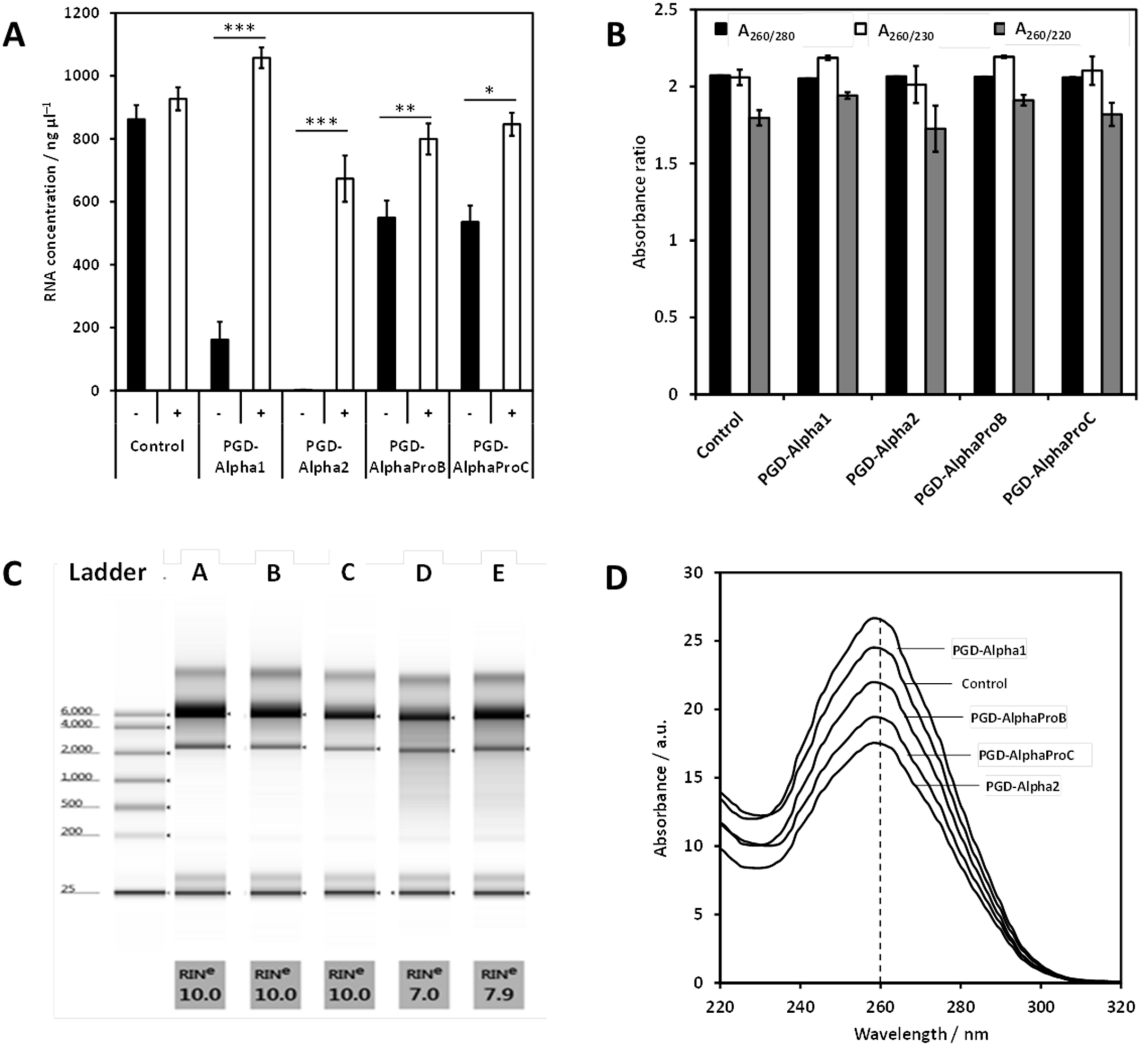
**A)** Concentration of RNA extracted from HEK293 cells encapsulated in the four peptide hydrogels, either with (+) or without (-) enzymatic pre-treatment. Three independent samples were measured with the data shown as mean ± SEM. Cells suspended in PBS were used as a positive control. The mean values were compared using an unpaired t-test,. ***, P ≤ 0.001; **, P ≤ 0.01; *, P ≤ 0.05. **B)** A_260_/A_230_ and A_260_/A_280_ ratios for RNA samples isolated from four different peptide hydrogels and the cell-only control following enzymatic digestion. Three independent samples were measured with the data shown as mean ± SEM. **C)** Representative electrophoresis traces and corresponding RIN values obtained for the total RNA extracted from cells encapsulated in the four peptide hydrogels following enzymatic digestion: cell-only control (A), PGD-Alpha1 (B), PGD-Alpha2 (C), PGD-AlphaProB (D) and PGD-AlphaProC (E). **D)** Representative UV spectra for RNA samples extracted following enzymatic digestion.

Five commercially available enzymes / enzyme mixtures: papain, pronase, proteinase K, thermolysin and trypsin, were tested for their ability to digest each of the four peptide hydrogels. The fraction of non-degraded peptide monomer was estimated after 5 minutes of digestion (Fig 5) and was observed to vary depending on the enzyme and hydrogel used. For PGD-Alpha2 and PGD-AlphaProB all five enzyme solutions resulted in ~ 20 to 30% of the peptide monomers being degraded. For PGD-Alpha1, the use of pronase and thermolysin seemed to result in a large amount of peptide monomer being degraded: ~ 100 and 70%, respectively. Similarly, PGD-AlphaProC was also more prone to degradation by both pronase and thermolysin: ~ 90 and 50%, respectively. Enzymatic degradation of peptide will depend on a number of factors including: the exact peptide sequence, its solubility, and the enzyme affinity for degrading that specific sequence. It is also known that the assembly of peptides into β-sheet fibrils provides protection from proteolytic degradation [35] and therefore the stability of the fibrillar aggregate will also affect the enzymatic degradation of that peptide. Understanding how proteolytic enzymes differentially degrade each peptide sequence is beyond the scope of this study. Nevertheless, from the results obtained, pronase was selected for its ability to significantly degrade PGD-Alpha1 and PGD-AlphaProC peptides; while showing similar degradation efficiency to the other enzymes for PGD-Alpha2 and PGD-AlphaProB. The RNeasy MK column-based extraction method was also chosen since it was more effective at producing RNA of high purity than the TRI Reagent method, as shown in the previous section. Fig 6A shows that by first enzymatically digesting the hydrogels with pronase it was possible to extract significantly higher levels of RNA from all four peptide hydrogels. The concentration of RNA obtained (based on A_260_ absorbance values–Fig 6D) from encapsulated cells was comparable to the cell-only control, except for PGD-Alpha2 for which a slightly lower yield was obtained. When comparing the concentration of RNA obtained with and without the use of pronase pre-treatment, the greatest improvement was seen with PGD-Alpha1 and PGD-Alpha2. In fact, RNA could not be extracted from cells encapsulated in PGD-Alpha2 using the RNeasy MK method without the use of pronase pre-treatment. The exact mechanism of how pronase improves the extraction of the RNA beyond the degradation of the peptide is complex, it should be kept in mind that following digestion, the lysis buffer is added and the enzyme is still present during homogenisation. It is therefore likely that further degradation occurs. In addition specific interactions between the enzyme(s) and the peptide / peptide fibres may also contribute to improving RNA extraction by inhibiting peptide fibres—RNA interactions. In addition, all samples of RNA extracted, after enzymatic hydrogel digestion, were found to be of high purity, comparable to the control (Fig 6B).

The RNA integrity was once again assessed by electrophoresis (Fig 6C). For all four hydrogels clear separation profiles, similar to the control, composed of intact 28S and 18S rRNA bands were obtained. For PGD-Alpha1 and PGD-Alpha2 a RIN value of 10 was assigned indicating that the RNA extracted was intact. For PGD-AlphaProB and PGD-AlphaProC, lower associated RIN values of 7.4 ± 0.2 and 8.2 ± 0.5 were assigned, respectively. However, these values were still found to be within the range expected for intact RNA (> 7), and above 5—a RIN value stated within the literature as a threshold for gaining reliable PCR results.[36] It should also be noted that the enzymatic pre-treatment of the cell-only control did not affect the yield of RNA obtained; nor its purity or integrity (comparison of results obtained for controls in Figs 3 and 6).

Finally the suitability of the RNA isolated from these hydrogels, following enzymatic digestion, for RT-qPCR was evaluated by comparing the expression levels of five common housekeeping genes encoding: glyceraldehyde 3-phosphate dehydrogenase (GAPDH), ribosomal protein L13a (RPL13A), β-actin (ACTB), β2 microglobulin (B2M) and 18S ribosomal RNA (RRN18s).

**Fig 7:**
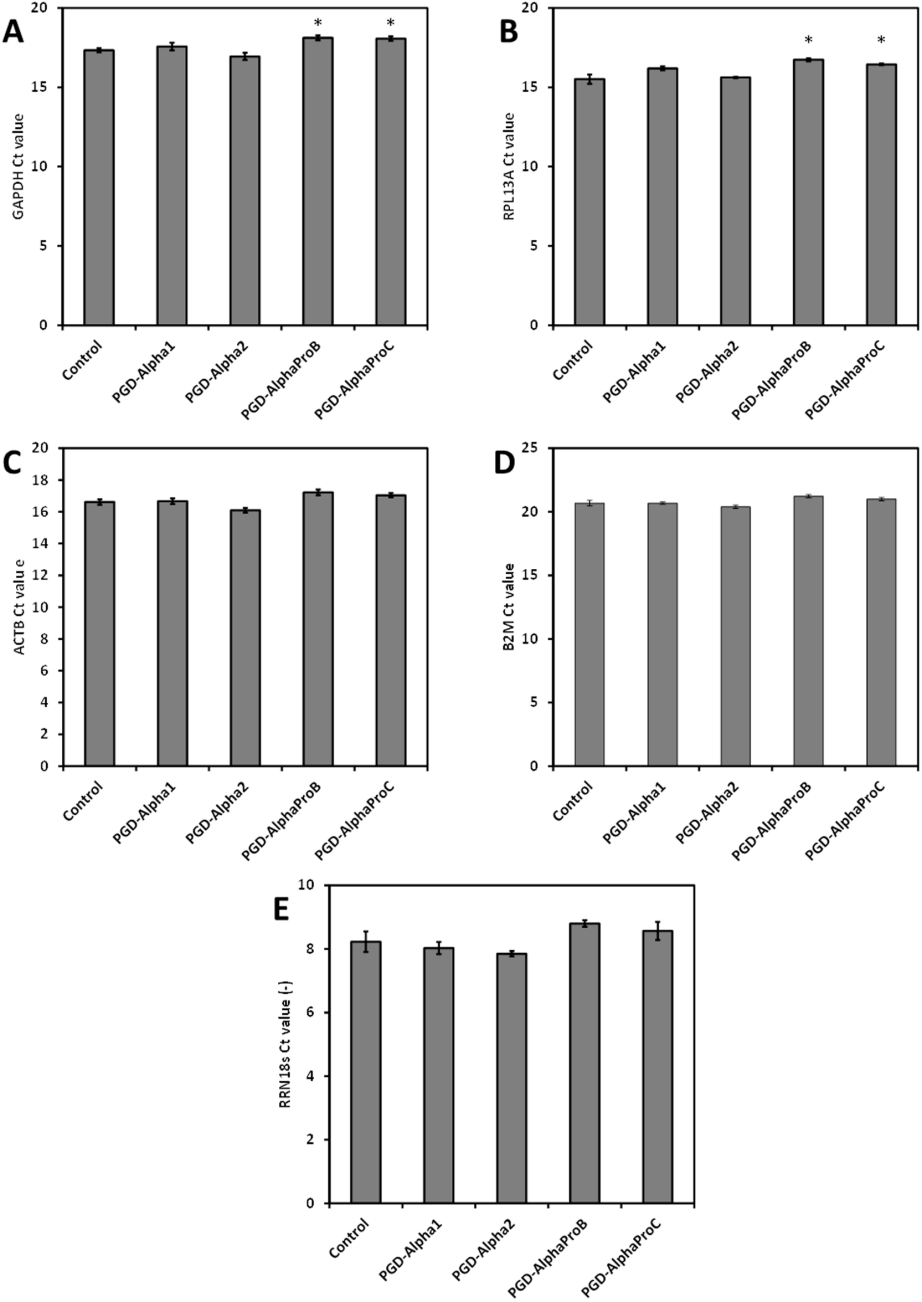
Ct values obtained for RT-qPCR performed using RNA extracted using RNeasy MK method from the four peptide hydrogels pre-treated with pronase enzyme solution and the cell-only control as a template. RNA extracted was used as templates for the amplification of five housekeeping genes: GAPDH (A), RPL13A (B), ACTB (C), B2M (D) and RRN18s (E) commonly expressed in HEK293 cells. The cycle threshold (Ct) values are presented as the mean ± SEM for three independent samples measured in triplicate. *, P < 0.05.

All five primer sets produced equally low Ct values for the control and each of the four peptide hydrogels (Fig 7). The melting temperatures of the RT-qPCR products were of the expected size and thereby validated that only the desired amplicon was detected. For four of the primer sets (GAPDH, RPL13A, ACTB and B2M) Ct values between 15 and 21 were observed, showing little difference between the amplification efficiency of RNA extracted from cells within each of the peptide hydrogels and the cell-only control. RRN18s is more abundantly expressed and as a result produced much lower Ct values ~ 8 (Fig 7E). Again no significant differences in Ct values were observed between the control and the hydrogels. These results clearly show that using the column-based extraction method and an enzymatic digestion pre-treatment, a high yield of pure and intact RNA can be extracted from these peptide hydrogels, which is suitable for down-stream analysis using RT-qPCR.

## Conclusions

We have investigated the effect of RNA-peptide interactions on RNA extraction protocols. We have shown that RNA interactions with peptide fibrils interfere with the extraction process, rather than molecular complexing of RNA and peptide monomer. Moreover, the amount of RNA extracted was critical to afford good quality RT-qPCR data. However, the contamination of RNA by small amounts of peptide carried through the extraction process did not interfere with the enzymes involved in cDNA synthesis nor RT-qPCR. Our results also show that methods based on solid-state binding of RNA are more suited to the extraction of RNA from β-sheet forming self-assembling peptide hydrogels, for this strong binding competes directly with the RNA-peptide fibril interactions. Pre-digestion of these hydrogels using a broad spectrum enzyme—pronase—was shown to significantly improve the extracted RNA yields for all four hydrogel formulations and makes this approach potentially applicable more universally across peptide-based hydrogels. The ability to extract high yields of high purity RNA is a key step towards the general use of these materials in the biological and medical fields and therefore to the fulfilment of their full potential.
